# ApoJ and apoL1 as novel determinants of MASH: a cross-sectional study

**DOI:** 10.1186/s12944-025-02733-0

**Published:** 2025-10-14

**Authors:** Zichun Cai, Souad Najib, María A. Núñez-Sánchez, María A. Martínez-Sánchez, Carmen García-Melgares, Nathalie Viguerie, Joana Rossell, Josep Julve, Mikaël Croyal, Arsênio Rodrigues Oliveira, Carlos M. Martínez, Sébastien J. Dumas, Annelise Genoux, María D. Frutos, Bruno Ramos-Molina, Laurent O. Martinez

**Affiliations:** 1 Université de Toulouse, Inserm, Institut des Maladies Métaboliques et Cardiovasculaires (I2MC), 1 Avenue du Pr. Jean Poulhès BP 84225, UMR1297, Toulouse, 31432 France; 2 IHU HealthAge, Toulouse, France; 3 Obesity, Diabetes, and Metabolism Laboratory, Biomedical Research Institute of Murcia (IMIB), Murcia, 30120 Spain; 4 Group of Endocrinology, Diabetes and Nutrition, Institut de Recerca Sant Pau, Barcelona, 08041 Spain; 5 CIBER of Diabetes and Associated Metabolic Diseases (CIBERDEM), Instituto de Salud Carlos III, Madrid, 28029 Spain; 6 Nantes Université, CNRS, INSERM, L’institut du Thorax, Nantes, 44000 France; 7 Nantes Université, CHU Nantes, CNRS, INSERM, BioCore, US16, SFR Bonamy, Nantes, F-44000 France; 8 CRNH-Ouest Mass Spectrometry Core Facility, Nantes, 44000 France; 9 Experimental Pathology Platform, Biomedical Research Institute of Murcia (IMIB), Murcia, Spain; 10 Department of Anatomy and Comparative Pathology, Faculty of Veterinary Science, University of Murcia, Campus de Espinardo, Murcia, 30100 Spain; 11 Department of General and Digestive System Surgery, Virgen de La Arrixaca University Hospital, Murcia, 30120 Spain

**Keywords:** Metabolic dysfunction-associated steatohepatitis, Obesity, Apolipoprotein, Biomarkers, Diagnosis

## Abstract

**Background:**

Plasma apolipoproteins are linked to cardiometabolic dysfunctions, but their potential as biomarkers for metabolic dysfunction-associated steatohepatitis (MASH) remains underexplored.

**Methods:**

Plasma levels of 14 apolipoproteins (apoA-I, A-II, A-IV, B100, C-I, C-II, C-III, D, E, F, H, J, L1, M) were quantified using liquid chromatography–tandem mass spectrometry in a cross-sectional study of 148 individuals with obesity undergoing bariatric surgery. Based on liver histology, participants were categorized as non-MASH (*n* = 94; no liver alterations or simple steatosis, ≥ 5% intrahepatic fat) or MASH (*n* = 54; steatosis with ballooning and lobular inflammation, with or without fibrosis). Correlations with clinical and biochemical parameters were assessed via Spearman’s rank correlation, and associations with MASH were evaluated using logistic regression. Incremental predictive value beyond established risk factors was assessed through likelihood ratio tests (LRT), net reclassification improvement (NRI), and integrated discrimination improvement (IDI).

**Results:**

ApoC-III and apoL1 were significantly higher in MASH compared with non-MASH participants, while other apolipoproteins showed no group differences. Higher apoE, apoL1 and apoJ levels were associated with increased odds of MASH, independently of age and sex. Associations for apoL1 and apoJ remained significant after adjustment for diabetes, dyslipidemia, and hypertension, or for established MASH risk factors including insulin resistance, triglycerides, waist circumference, and the AST/ALT ratio. LRT analyses showed that apoJ (ΔDeviance = 4.085, *p* = 0.043) and apoL1 (ΔDeviance = 3.954, *p* = 0.047) each improved model fit, with their combination providing additional improvement (ΔDeviance = 7.534, *p* = 0.023). NRI analysis indicated that the combination of apoJ and apoL1 provided the largest improvement (NRI total = 0.39, *p* = 0.026), mainly by correctly reclassifying non-MASH individuals (NRI non-event = 0.31, *p* = 0.0023). IDI was also greatest for the combination (IDI = 0.04, *p* = 0.034), indicating enhanced discrimination between MASH and non-MASH individuals. In an external cohort, the elevation of plasma apoJ in MASH was consistently replicated, whereas apoL1, apoC-III, and apoE showed no such pattern.

**Conclusions:**

Plasma apoJ and apoL1 may serve as potential biomarkers for diagnosing MASH in individuals with obesity, independent of traditional risk factors. Further validation in larger cohorts and mechanistic studies is warranted.

**Supplementary Information:**

The online version contains supplementary material available at 10.1186/s12944-025-02733-0.

## Introduction

Metabolic dysfunction-associated steatotic liver disease (MASLD) is a chronic progressive condition primarily characterized by abnormal triglyceride accumulation in the liver, known as simple hepatic steatosis [1]. Over time, MASLD might progress to metabolic dysfunction-associated steatohepatitis (MASH), characterized by hepatocyte ballooning and lobular inflammation, and, in severe cases, liver fibrosis, significantly increasing the risk of hepatocellular carcinoma and cardiovascular disease [2].

MASH is particularly prevalent among individuals with obesity, who may also present other metabolic traits such as type 2 diabetes (T2D), insulin resistance, and dyslipidemia. Despite the link between MASH and obesity, a subset of individuals with obesity appears metabolically healthy and has a lower prevalence of MASH, often limited to simple steatosis, although progression to MASH can still occur [3–5]. Thus, identifying circulating markers for MASH in individuals with obesity, independent of their metabolic status, is clinically relevant. Notably, while non-invasive methods like transient elastography and biomarker-based scoring systems assist in screening and monitoring, liver biopsy remains the gold standard for MASH diagnosis when precise staging is required [6]. Thus, novel non-invasive biomarkers are crucial to improve diagnostic accuracy and reduce reliance on invasive procedures.

Beyond the need for better biomarkers, pharmacological options for MASH remain limited. Resmetirom, a thyroid hormone receptor β-agonist, is the only specific approved therapy, yet only 30% of patients achieve MASH resolution without fibrosis progression [7]. Meanwhile, GLP-1 and GIP/GLP-1 receptor agonists, which show promising results in weight loss and MASH resolution [8], though their long-term effects remain under investigation [9]. Currently, the MASH management relies primarily on dietary and lifestyle interventions to promote weight loss through healthy eating, calorie reduction, and increasing physical activity. When these measures fail, bariatric surgery (BS) has been the primary option to treat patients with non-cirrhotic MASLD/MASH [10].

This underscores the need for alternative treatments and precision medicine approaches, including the identification of novel biomarkers. Given the complex etiology of MASLD/MASH—shaped by genetic predisposition (e.g., PNPLA3 variants), metabolic dysregulation, lifestyle factors, and environmental influences—targeted approaches are essential for effective disease management.

Apolipoproteins are multifunctional proteins that participate in lipoprotein assembly, structure, and metabolism, influencing lipid uptake through interaction with cell-surface receptor and regulating the activity of lipase and lipid-transfer enzymes [11]. Previous studies have associated plasma levels of apoB100 (the main LDL apolipoprotein), apoA-I (the main HDL apolipoprotein), apoC-III (a natural inhibitor of lipoprotein lipase), and apoF with MASLD [12–16]. However, other apolipoproteins with less well-characterized functions remain poorly investigated in the context of MASH diagnosis. In this cross-sectional study, we examined the associations between plasma concentrations of 14 apolipoproteins and MASH status in individuals with obesity undergoing bariatric surgery, with the aim of evaluating their potential utility as plasma-based biomarkers for early disease detection and risk stratification.

## Subjects and methods

### Study participants

**Fig. 1:**
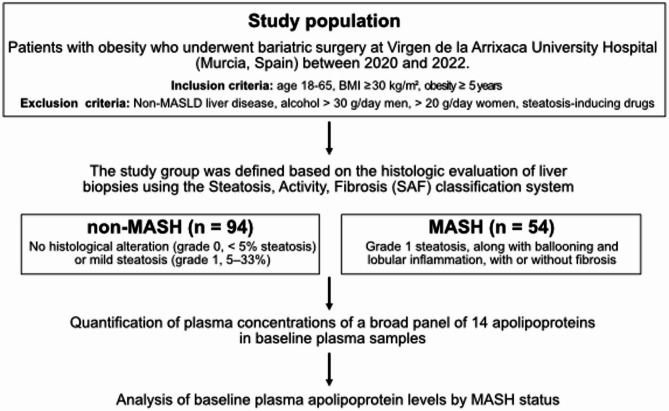
Study flowchart. The study population includes patients with obesity who underwent bariatric surgery and were classified as non-MASH (*n* = 94) or MASH (*n* = 54) based on liver histology. Plasma levels of 14 apolipoproteins were analyzed at baseline. Apolipoprotein levels were compared according to MASH status

The study workflow is illustrated in Fig. 1. The present study, referred to as the discovery cohort, included a total of 148 individuals with obesity who underwent BS (Roux-en-Y gastric bypass) at the Virgen de la Arrixaca University Hospital (Murcia, Spain) between 2020 and 2022. Inclusion criteria included a signed informed consent, age between 18 and 65 years, a body mass index (BMI) ≥ 30 kg/m^2^ with significant obesity-related comorbidities, and a duration of obesity of five years or more. Exclusion criteria included evidence of liver disease other than MASLD (including viral hepatitis, medication-related disorders, autoimmune disease, hepatocellular carcinoma, hemochromatosis, Wilson’s disease, familial/genetic causes), a previous history of excessive alcohol consumption (> 30 g daily for men and > 20 g daily for women), treatment with any drugs potentially causing steatosis (e.g. tamoxifen, amiodarone, and valproic acid), or subjects who declined to participate.

### Liver sample collection and histological analysis

Intraoperative wedge liver biopsies of at least 1cm^2^ were obtained from individuals who underwent BS. One section of the biopsy was rapidly frozen and stored at –80℃, whereas the other section was fixed in formalin and embedded in paraffin for histopathological evaluation. 5 μm sections of paraffin-embedded liver biopsies were stained using hematoxylin and eosin, Masson’s trichrome, periodic acid-Schiff, Perls, and reticulin staining. Pathologists from the Virgen de la Arrixaca University Hospital and the Experimental Pathology Unit of the Biomedical Research Institute of Murcia reviewed and scored all biopsies according to the steatosis, activity, and fibrosis (SAF) score, as previously described [17, 18].

### Study design

The study population was categorized into two groups based on the histopathological evaluation of liver biopsies using the SAF classification system [19]: (1) the non-MASH group, which included participants whose liverbiopsies showed no histologic alterations (grade 0, < 5 % steatosis) or mild steatosis (grade 1,5–33 %), and (2) the MASH group, which included participants whose liver biopsiesdemonstrated at least grade 1 steatosis, along with evidence of ballooning and lobularinflammation, with or without fibrosis.

Lifestyle risk factors included smoking, where an individual is considered a non-smoker if they have never smoked or are a former smoker who has not smoked for at least 5–10 years, and alcohol consumption, where an individual is considered a non-alcohol consumer if no alcoholic habits have been reported. Comorbidities included hypertension (systolic blood pressure [SBP] ≥ 140 mmHg, and/or diastolic blood pressure [DBP] ≥ 90 mmHg at rest, and/or antihypertensive treatment), diabetes (fasting plasma glucose [FPG] ≥ 126 mg/dL, and/or hypoglycaemic treatment) and dyslipidemia (LDL-C ≥ 160 mg/dL, and/or triglyceride levels ≥ 150 mg/dL, and/or use of lipid-lowering treatment).

### Biochemical analysis

Blood samples were obtained on the day of the surgery after a minimum 12-h overnight fast, and serum and plasma were obtained by centrifugation. Samples were anonymized and blinded for MASH status. The following parameters were measured using the Cobas Analyzer c702 (Roche): glucose, total cholesterol, high-density lipoprotein cholesterol (HDL-C), low-density lipoprotein cholesterol (LDL-C), triglycerides, alanine aminotransferase (ALT), and aspartate aminotransferase (AST) levels. Glycated hemoglobin (HbA_1C_) levels were determined using the glycohemoglobin analyzer HLC-723G8 (Tosoh Bioscience). Insulin levels were measured using the Cobas Analyzer e801 (Roche). Insulin resistance was assessed using the homeostasis model assessment of the insulin resistance index (HOMA-IR), calculated as insulin (µU/mL) x glucose (mmol/L)/22.5 [20].

### Apolipoprotein measurements.

Plasma concentrations of apolipoproteins (A-I, A-II, A-IV, B100, C-I, C-II, C-III, D, E, F, H, J, L1, and M) were determined by liquid chromatography-tandem mass spectrometry, as described previously [21]. Briefly, the apolipoproteins were quantified in 40-µL aliquots (EDTA plasma) using trypsin proteolysis and the subsequent analysis of proteotypic peptides. The intra- and inter-assay variabilities were measured and did not exceed 9.4% [21].

### RNA purification and qPCR analysis

Liver biopsies were collected during surgery and further preserved at –80℃ in RNAlater (Sigma) until analysis. Total RNA was extracted and purified using TRIzol reagent (Life Technologies) and GeneJET RNA Purification kit (Thermo Scientific). One microgram of purified RNA was reverse transcribed using the High-Capacity RNA-to-cDNA kit (Applied Biosystems) according to the manufacturer’s instructions. For gene transcription quantification, qPCR amplification was performed using the Power SYBR Green Master mix (Applied Biosystems) on a Fast 7500 Real-Time instrument (Applied Biosystems), and relative transcription levels were calculated using the 2^ − ΔΔCt method, with 18S rRNA as a housekeeping gene.

### Candidate apolipoprotein analysis in an external cohort

Relative quantification of plasma apoL1, apoJ, apoC-III and apoE were obtained from a mass spectrometry-based proteomics analysis of plasma samples from an external cohort of 160 obese individuals undergoing bariatric surgery [22]. Based on liver histology, participants were stratified into two groups: non-MASH (no liver alterations or simple steatosis; *n* = 127) and MASH (*n* = 33). Plasma proteomics data were generated using the Small Protein Enrichment Assay (SPEA) workflow, with protein abundance values determined from proteolytic peptide intensity following Log₂ transformation and mean normalization, as reported in Supplementary Table S6 of the reference study [22]. Raw data are publicly available via the ProteomeXchange Consortium under accession number PXD052798.

### Gene and protein nomenclature

Gene and protein names are based on gene names and were capitalized (Hugo Gene Nomenclature Committee, https://www.genenames.org/about/guidelines/).

### Statistical analysis

All categorical parameters were expressed as the number (%) and tested by Pearson’s Chi-squared test. All quantitative parameters were expressed as the mean ± standard deviation (SD) and tested using two-tailed unpaired Student’s t test unless otherwise specified. When the distribution was considered as skewed, parameters were expressed as the median (25th percentile; 75th percentile) and tested by Wilcoxon rank sum test. The Benjamini–Hochberg procedure with false discovery rate (FDR) was used to control for multiple comparisons when appropriate.

The correlation between the baseline characteristics was studied as a cross-sectional study. Spearman’s correlation coefficients (*r*_*s*_) were calculated between plasma apolipoprotein concentrations and the clinical characteristics, glucose homeostasis, and lipid values with *p* < 0.05 considered statistically significant.

Multivariable regression models were applied with the following adjustments: adjusted for age and sex (model 1), adjusted for age, sex, HOMA-IR, triglycerides, waist circumference, and AST/ALT ratio (model 2), and adjusted for age, sex, hypertension, diabetes and dyslipidemia (model 3). Lack of multicollinearity between apolipoproteins and the adjustment variables was assessed by examining the variance inflation factors.

To evaluate the incremental predictive value of the candidate biomarkers, likelihood ratio tests (LRT), net reclassification improvement (NRI), and integrated discrimination improvement (IDI) were performed. The LRT was used to assess whether the extended models incorporating apoJ and/or apoL1 improved overall model fit compared with the core risk factor model. Category-free NRI and IDI were calculated to quantify the improvement in individual risk reclassification and overall discriminative ability, respectively, when the candidate biomarkers were added to the baseline model.

All analyses were performed using R software version 4.0.0 [23], and the R scripts are available upon request.

## Results

### Characteristics of the study population

Baseline clinical and biochemical characteristics for the entire cohort, as well as for two groups categorized by MASH status (non-MASH vs MASH), are summarized in Table 1. The overall population consisted of middle-aged individuals (46 ± 11 years), with severe obesity (BMI: 43.4 ± 6.0 kg/m^2^), predominantly women (75%), and exhibited insulin resistance (HOMA-IR: 3.0 [1.6; 4.8]) [24]. Other glucose metabolism parameters, including FPG, insulin and HbA_1c_ were mostly normal or near abnormal thresholds, while plasma lipids, such as triglycerides and LDL-C, were slightly elevated, likely reflecting partial treatment within the population. Accordingly, 72.3% had dyslipidemia, but only 29.1% received lipid-lowering drugs, while 45.9% had diabetes with 43.9% on anti-diabetic medications (Table 1). The prevalence of hypertension was high (72.3%), yet only 35.8% of affected individuals received antihypertensive treatment. Overall, solely 15.5% of the study population had neither a diagnosis nor treatment for comorbidities.

**Table 1:** Sociodemographic, clinical and biological characteristics of the study population according to MASH status

**Characteristics**	**Whole population** (*n* = 148)	**Non-MASH** (*n*_1_ = 94)	**MASH** (n_2_ = 54)	***p*-value**	**N/A** (n_1_, n_2_)
***Age***	46 ± 11	45 ± 11	48 ± 10	0.078^1^	(0,0)
***Gender***				**0.030**^2^	(0,0)
**Women, n (%)**	111 (75.0)	76 (80.9)	35 (64.8)		
**Men, n (%)**	37 (25.0)	18 (19.1)	19 (35.2)		
***Smoking***				0.625^2^	(1,0)
No, n (%)	86 (58.5)	53 (57.0)	33 (61.1)		
Yes, n (%)	61 (41.5)	40 (43.0)	21 (38.9)		
***Alcohol consumption***				0.279^2^	(1,0)
No, n (%)	133 (90.5)	86 (92.5)	47 (87.0)		
Yes, n (%)	14 (9.5)	7 (7.5)	7 (13.0)		
***Anthropometric measures***					
BMI, kg/m^2^	43.4 ± 6.0	43.5 ± 5.6	43.3 ± 6.8	0.849^1^	(0,0)
Waist circumference, cm	124 [117; 134]	124 [117; 132]	127 [118; 139]	0.230^3^	(1,0)
***Glucose metabolism***					
**FPG**, mg/dL	95 [84; 104]	92 [84; 100]	102 [89; 123]	**< 0.001**^3^	(0,0)
**Hb1Ac**, %	5.70 [5.40; 6.10]	5.60 [5.30; 5.95]	5.80 [5.60; 6.70]	**0.001**^3^	(3,0)
**Insulin,** μUI/mL	12 [7; 19]	12 (7, 18)	15 [9; 27]	**0.017**^3^	(1,0)
**HOMA-IR**	3.0 [1.6; 4.8]	2.7 [1.5; 4.0]	3.7 [2.2; 7.7]	**0.002**^3^	(0,0)
***Lipid metabolism***					
Total cholesterol, mg/dL	163 ± 31	163 ± 31	163 ± 32	0.949^1^	(0,0)
**Triglycerides**, mg/dL	167 [125; 212]	155 [121; 206]	189 [154; 229]	**0.035**^3^	(1,0)
**HDL-C**, mg/dL	40 [34; 49]	42 [36; 50]	38 [33; 44]	**0.050**^3^	(1,0)
LDL-C, mg/dL	87 ± 29	88 ± 28	85 ± 31	0.617^1^	(7,3)
***Transaminases***					
**AST**, U/L	18 [15; 23]	17 [14; 20]	22 [18; 28]	**< 0.001**^3^	(5,5)
**ALT**, U/L	19 [14; 28]	16 [12; 22]	24 [19; 40]	**< 0.001**^3^	(2,0)
**AST/ALT**	0.95 [0.78; 1.16]	1.06 [0.87; 1.29]	0.90 [0.69; 1.00]	**< 0.001**^3^	(6,5)
***Blood pressure***					
SBP, mmHg	140 [130; 150]	139 [126; 149]	141 [130; 150]	0.112^3^	(0,0)
DBP, mmHg	85 ± 12	86 ± 12	84 ± 11	0.553^1^	(0,0)
***Comorbidities and treatments***					
**T2D**, n (%)^4^	68 (45.9)	33 (35.1)	35 (64.8)	**< 0.001**^2^	(0,0)
**Treatment T2D**, n (%)	65 (43.9)	32 (34.0)	33 (61.1)	**0.001**^2^	(0,0)
**Dyslipidemia**, n (%)^5^	107 (72.3)	59(62.8)	48 (88.9)	**0.001**^2^	(2,0)
**Treatment dyslipidemia**, n (%)	43 (29.1)	22 (23.4)	21 (38.9)	**0.046**^2^	(0,0)
Hypertension, n (%)^6^	107 (72.3)	67 (71.3)	40 (74.1)	0.714^2^	(0,0)
Treatment hypertension, n (%)	53 (35.8)	32 (34.0)	21 (38.9)	0.554^2^	(0,0)
None, n (%)	23 (15.5)	17 (18.1)	6 (11.1)	0.260^2^	(0,0)

Categorical parameters are expressed as the number of individuals (%). Quantitative parameters are expressed as the mean ± SD for Gaussian distribution or as the median [25th percentile; 75th percentile] for non-Gaussian distribution. The difference between the two groups (non-MASH versus MASH) was analyzed using the following statistical tests

^1^Two-tailed unpaired Student’s t-test

^2^Pearson's Chi-squared test, or

^3^Wilcoxon rank sum test. Comorbidities were defined as follows

^4^Diabetes: fasting plasma glucose ≥ 126 mg/dL and/or treatment

^5^Dyslipidemia: LDL-C ≥ 160 mg/dL and/or triglycerides (TG) ≥ 150 mg/dL or treatment

^6^Hypertension: systolic blood pressure ≥ 140 mmHg or diastolic blood pressure ≥ 90 mmHg or treatment; ^7^None: no diabetes, no dyslipidemia, nor hypertension. ALT, alanine aminotransferase; AST, aspartate aminotransferase; DBP, diastolic blood pressure; FPG, fasting plasma glucose; HbA1c, glycated hemoglobin; HOMA-IR, homeostatic model assessment of insulin resistance; N/A, not available; SBP, systolic blood pressure; T2D, type 2 diabetes

Significant differences were observed between the non-MASH and MASH groups. Specifically, the diagnosis of MASH was associated with a marked difference in sex distribution, with women being significantly less represented in the MASH group (64.8% versus 80.9% in the non-MASH group, *p* = 0.03). Individuals with MASH were more insulin resistant, as documented by higher HOMA-IR values compared to the non-MASH (*p* = 0.002), along with significantly higher level of FPG, insulin, and HbA_1c_. Additionally, triglycerides were significantly higher, and HDL-C levels were significantly lower in the MASH group compared to the non-MASH group. Consistent with these metabolic differences, individuals with MASH had a higher prevalence of diabetes and dyslipidemia, but showed no difference in the prevalence of hypertension. Furthermore, individuals with MASH had higher levels of transaminases (AST and ALT), accompanied by a lower AST/ALT ratio (*p* < 0.001). No significant differences were observed between groups in terms of anthropometric measures (BMI, waist circumference) and lifestyle risk factors such as smoking and alcohol consumption.

### Plasma concentration of apolipoproteins by MASH status

Baseline concentrations of 14 plasma apolipoproteins, stratified by MASH status, are presented in Table 2. Plasma levels of apoC-III and apoL1 were nominally higher in the MASH group compared with the non-MASH group (*p* = 0.038 and *p* = 0.025, respectively). Levels of apoC-I, apoC-II, apoE, and apoJ were also higher in the MASH group (*p* < 0.10). After correction for multiple testing, all six apolipoproteins (apoC-I, apoC-II, apoE, apoJ, apoC-III, and apoL1) showed an FDR-adjusted *p*-value (q) of 0.217 (Supplementary Table 3). In contrast, apoA-I, apoA-II, apoA-IV, apoB100, apoD, apoF, apoH, and apoM did not differ nominally between groups (*p* > 0.10).

**Table 2:** Plasma concentration of apolipoproteins according to MASH status

**Apolipoproteins** (mg/dL)	**Whole population** (*n* = 148)	**Non-MASH** (n_1_ = 94)	**MASH** (n_2_ = 54)	***p*-value**	**N/A** (n_1_, n_2_)
ApoA-I	120 ± 28	122 ± 30	115 ± 23	0.116^1^	(0,0)
ApoA-II	18.1 ± 5.3	18.2 ± 5.3	17.9 ± 5.5	0.768^1^	(0,0)
ApoA-IV	11.6 ± 5.6	11.5 ± 5.5	11.8 ± 5.9	0.720^1^	(0,0)
ApoB100	89 ± 42	88 ± 41	92 ± 43	0.548^1^	(0,0)
ApoC-I	2.22 ± 0.78	2.14 ± 0.77	2.37 ± 0.77	0.083^1^	(0,0)
ApoC-II	3.23 ± 2.86	2.91 ± 2.62	3.80 ± 3.18	0.083^1^	(0,0)
**ApoC-III**	**5.41 ± 1.79**	**5.16 ± 1.58**	**5.85 ± 2.06**	**0.038**^1^	(0,0)
ApoD	11.5 ± 4.0	11.3 ± 4.0	11.9 ± 4.1	0.391^1^	(0,0)
ApoE	5.54 ± 1.78	5.36 ± 1.90	5.85 ± 1.49	0.090^1^	(0,0)
ApoF	1.60 ± 0.38	1.57 ± 0.39	1.65 ± 0.37	0.189^1^	(0,0)
ApoH	4.70 ± 2.17	4.71 ± 2.32	4.67 ± 1.89	0.904^1^	(0,0)
ApoJ	9.8 ± 5.5	9.2 ± 4.8	10.9 ± 6.4	0.093^1^	(0,0)
**ApoL1**	**2.40 ± 1.05**	**2.25 ± 0.98**	**2.67 ± 1.13**	**0.025**^1^	**(0,0)**
ApoM	2.27 ± 0.62	2.22 ± 0.66	2.36 ± 0.54	0.163^1^	(0,0)

Data are expressed as the mean ± SD

^1^Two-tailed unpaired Student’s t-test

N/A, not available

### Association between plasma apolipoprotein levels and biochemical parameters

**Fig. 2:**
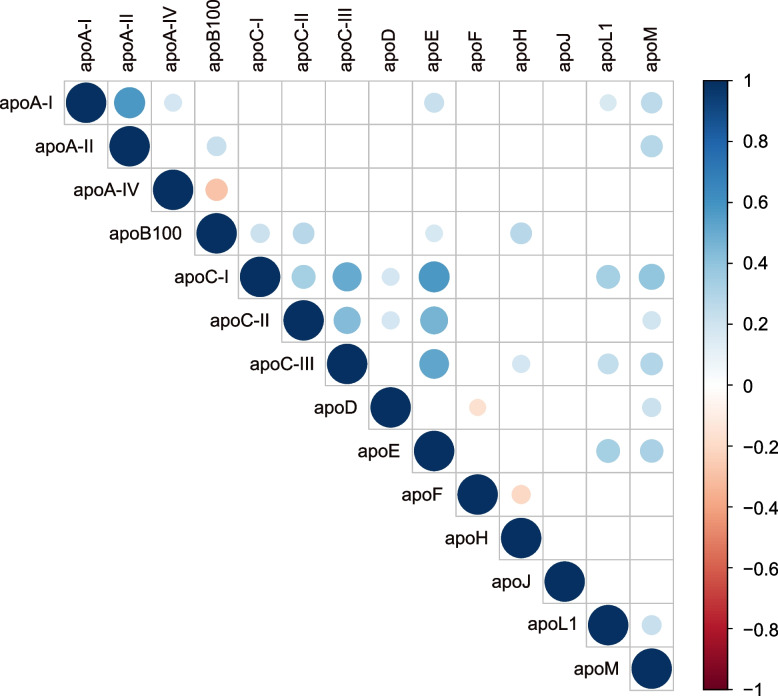
Spearman correlations between plasma apolipoprotein concentrations. Plasma apolipoproteins were measured in the whole study population (*n* = 148). Size and color, according to the color scale detailed on the right, represent the strength of the correlation intensity between variables, with Spearman’s correlation coefficient (*rs*) ranging from −1 to + 1. Only correlations significant at the 5% threshold are shown. Non-significant correlations are indicated by empty boxes. Blue dot, positive correlation; red dot, negative correlation

**Fig. 3:**
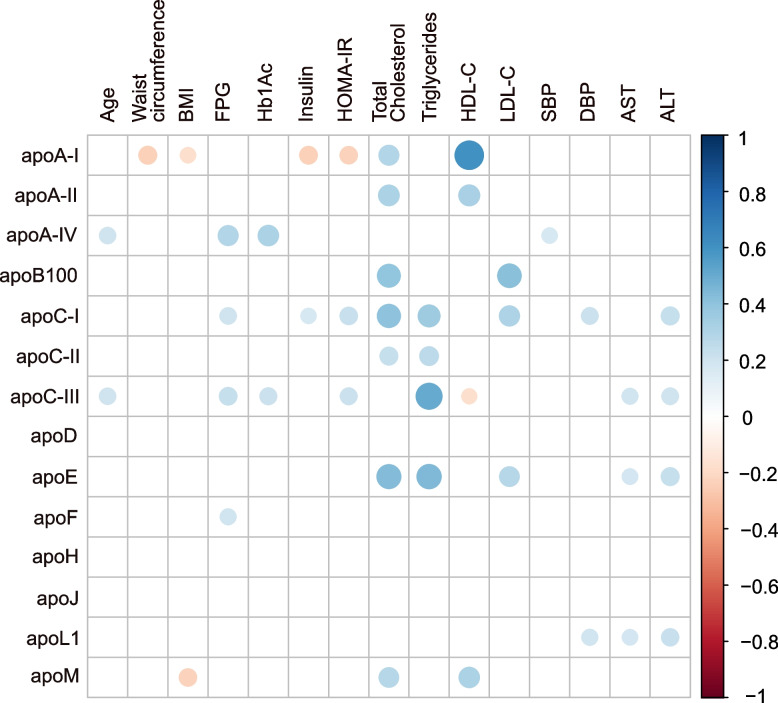
Spearman correlations between plasma apolipoprotein concentrations and bioclinical variables. Plasma apolipoproteins were measured in the whole study population (*n* = 148). Size and color, according to the color scale detailed on the right, represent the strength of the correlation intensity between variables, with Spearman’s correlation coefficient (*r*_*s*_) ranging from −1 to + 1. Only correlations significant at the 5% threshold are shown. Non-significant correlations are indicated by empty boxes. Blue dot, positive correlation; red dot, negative correlation. ALT, alanine aminotransferase; AST, aspartate aminotransferase; BMI, body mass index; DBP, diastolic blood pressure; FPG, fasting plasma glucose; HbA_1c_, glycated hemoglobin; SBP, systolic blood pressure

For the study population, Spearman’s rank correlation coefficients (*r*_*s*_) were calculated to assess associations among plasma apolipoproteins (Fig. 2 and Supplementary Table 1), as well as between apolipoproteins and biochemical parameters at baseline (Fig. 3 and Supplementary Table 2).

Many apolipoproteins were intercorrelated, consistent with their shared transport by lipoproteins [25] (Fig. 2). Specifically, apoA-I, the primary HDL apolipoprotein, showed positive correlations with apoA-II, apoE, apoL1, and apoM. Similarly, significant positive intercorrelations were observed among apoB100—a major protein constituent of VLDL and LDL—apoE, apoC-I, apoC-II, and apoC-III. Among other apolipoproteins exhibiting multiple correlations, apoL1 was positively correlated with apoA-I, apoC-I, apoC-III, apoE and apoM. ApoM displayed a similar correlation profile to apoL1, with additional correlations to apoA-II, apoC-II, and apoD, consistent with its transport by both HDL and LDL [26]. In contrast, apoA-IV was positively correlated only with apoA-I and negatively correlated with apoB100, while apoF was negatively correlated only with apoD and apoH. Additionally, apoD was positively correlated with both apoC-I and apoC-II, while apoH showed positive correlations with apoB100 and apoC-III. Notably, apoJ was the only apolipoprotein that did not show any correlation with the other apolipoproteins (Fig. 2).

As expected, most plasma apolipoproteins were associated with lipid levels (Fig. 3). Half of the measured apolipoproteins, including apoA-I, apoA-II, apoB100, apoC-I, apoC-II, apoE, and apoM, showed a positive correlation with total cholesterol. ApoB100, apoC-I and apoE were positively associated with LDL-C, while apoC-III exhibited a negative correlation with HDL-C. In contrast, apoA-I, apoA-II and apoM were positively correlated with HDL-C. Additionally, apoC-I, apoC-II, apoC-III and apoE displayed a positive correlation with triglycerides.

Regarding glycemic parameters (Fig. 3), apoA-IV, apoC-I, apoC-III, and apoF levels were positively correlated with FPG. Additionally, apoC-I was positively correlated with insulin, and both apoC-I and apoC-III showed significant positive correlations with HOMA-IR. ApoA-IV and apoC-III were also positively correlated with HbA_1c_.

ApoC-III, apoE and apoL1 exhibited positive correlations with plasma transaminase levels (AST and ALT). ApoA-IV and apoC-III were positively correlated with age, whereas apoA-I was negatively correlated with BMI and waist circumference. Furthermore, apoA-IV was positively associated with SBP, while apoC-I and apoL1 were positively associated with DBP. Noteworthily, no correlation was observed between apoH or apoJ and any of the measured biochemical parameters (Fig. 3).

### Plasma apolipoprotein levels and MASH diagnosis

**Fig. 4:**
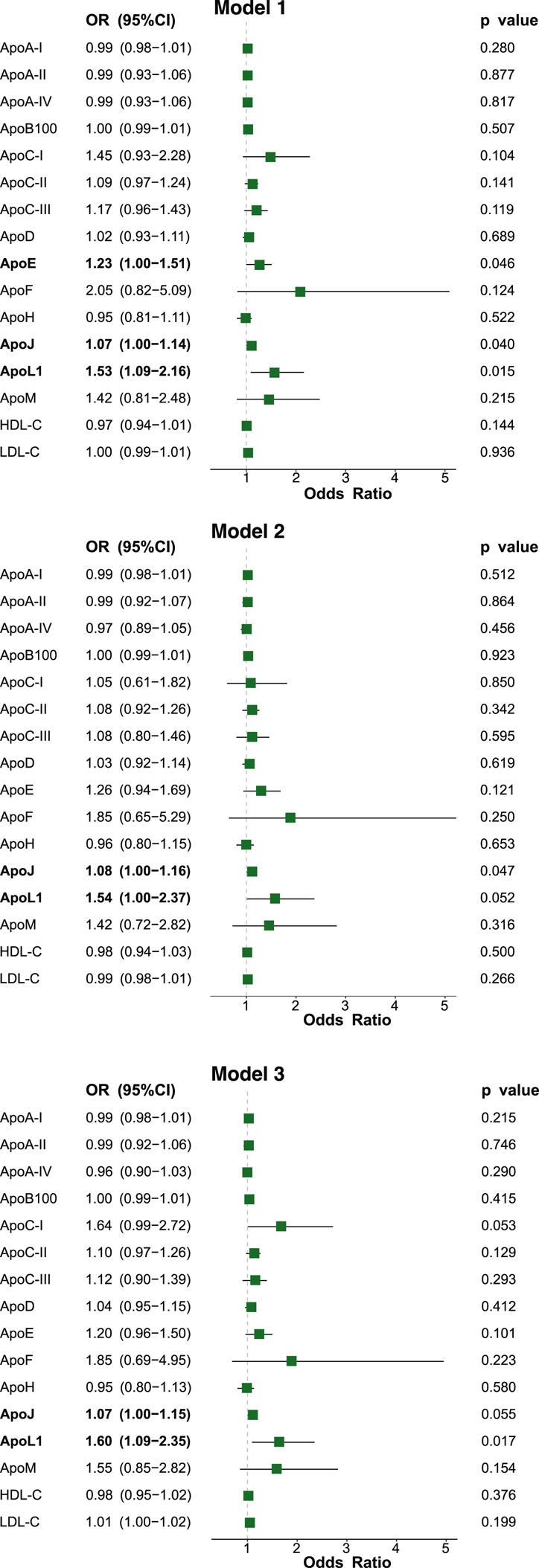
Multivariable logistic regression models assessing MASH status with plasma apolipoprotein levels as independent variables. Model 1: Adjusted for age and sex. Model 2: Model 1 + HOMA-IR, plasma triglyceride levels, waist circumference, and the AST/ALT ratio. Model 3: Model 1 + diabetes, dyslipidemia, and hypertension. The 95% confidence interval (95% CI), and p-value associated with each odds ratio (OR) are reported. Dyslipidemia was defined as LDL-C ≥ 160 mg/dL and/or triglyceride levels ≥ 150 mg/dL and/or use of lipid-lowering treatment. Hypertension was defined as systolic blood pressure (SBP) ≥ 140 mmHg and/or diastolic blood pressure (DBP) ≥ 90 mmHg at rest, and/or antihypertensive treatment. Diabetes was defined as fasting plasma glucose (FPG) ≥ 126 mg/dL and/or hypoglycaemic treatment. ALT, alanine aminotransferase; AST, aspartate aminotransferase; HOMA-IR, Homeostatic Model Assessment of Insulin Resistance

The results of multivariable logistic regression analyses examining plasma apolipoproteins, HDL-C, and LDL-C levels in relation to MASH status are presented in Fig. 4.

In a model adjusted for age and sex (Model 1), plasma apoE, apoJ, and apoL1 were positively associated with a diagnosis of MASH. The respective odds ratios (OR) per 1-SD increase were as follows: apoE (OR = 1.23, 95% CI [1.00–1.51], *p* = 0.046), apoJ (OR = 1.07, 95% CI [1.00–1.14], *p* = 0.040), and apoL1 (OR = 1.53, 95% CI [1.09–2.16], *p* = 0.015).

These associations persisted for apoJ and apoL1 after further adjustment for traditional MASH risk factors, including HOMA-IR, plasma triglyceride levels, waist circumference, and the AST/ALT ratio (Model 2: apoJ, OR = 1.08, 95% CI [1.00–1.16], *p* = 0.047; apoL1, OR = 1.54, 95% CI [1.00–2.37], *p* = 0.052), as well as for comorbidities such as diabetes, dyslipidemia, and hypertension (Model 3: apoJ, OR = 1.07, 95% CI [1.00–1.15], *p* = 0.055; apoL1, OR = 1.60, 95% CI [1.09–2.35], *p* = 0.017).

**Fig. 5:**
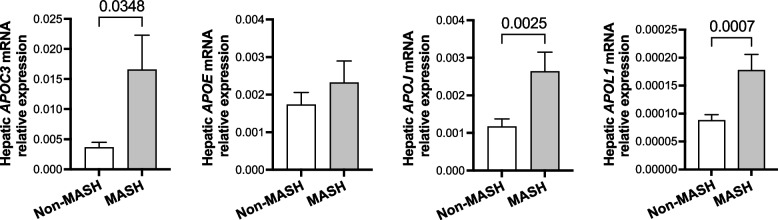
*APOC3*, APOE, *APOJ*, and *APOL1* mRNA expression levels in liver based on the MASH status of individuals with obesity. Subjects who underwent liver biopsy (*n* = 141) during bariatric surgery were stratified into two groups: those without liver disease or with simple steatosis (non-MASH, n = 87) and those with MASH (n = 54). Data are presented as mean ± SEM. Two-tailed unpaired Student’s *t*-test was used for MASH status comparison, and *p* values < 0.05 are reported

**Fig. 6:**
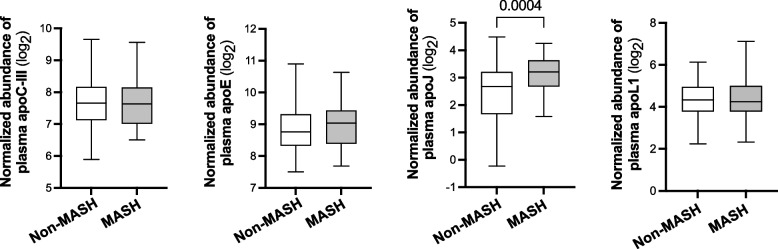
Signal intensity of apoC-III, apoE, apoJ, and apoL1 peptides in plasma from an external cohort of individuals with obesity undergoing bariatric surgery, stratified into two groups: those without liver disease or with simple steatosis (non-MASH, *n* = 127) and those with MASH (*n* = 33). Data are presented as median with interquartile range (IQR); whiskers indicate minimum and maximum values. Comparisons between groups were performed using a two-tailed unpaired Student’s t-test, and *p* values < 0.05 are reported

Notably, among plasma apolipoproteins, those with differential concentration based on MASH status (apoC-III and apoL1, Table 2) and/or those associated with MASH in multivariable logistic regression (apoE, apoJ, and apoL1, Fig. 4) were further examined at the transcriptional level by measuring their corresponding mRNA expression in the liver samples from the current cohort (Fig. 5), as well as the plasma levels in an independent external cohort of obese individuals, similarly classified as non-MASH (no liver alterations or simple steatosis) or MASH [22] (Fig. 6). As shown in Fig. 5, hepatic mRNA levels of *APOC3*, *APOJ*, and *APOL1*—but not *APOE*—were higher in individuals with obesity who were histologically diagnosed with MASH compared to those without MASH (*p* = 0.0348, 0.0025, and 0.007, respectively). In the external cohort, plasma levels of apoJ were also higher in the MASH group compared to the non-MASH group (*p* = 0.004, Fig. 6), whereas no differences were observed for apoL1, apoC-III, or apoE**.**

### Incremental predictive values of apoJ and apoL1 for MASH diagnosis beyond traditional risk factors

We evaluated whether apoJ and apoL1 improved prediction beyond a core risk factor model (age, sex, HOMA-IR, plasma triglycerides, waist circumference, and AST/ALT ratio) using likelihood ratio tests (LRT), net reclassification improvement (NRI), and integrated discrimination improvement (IDI) (Table 3).

**Table 3:** Incremental predictive values for apoJ and apoL1 for MASH diagnosis beyond clinical and biological risk factors

**Models**	**LRT Δ Deviance** (*p*-value)	**NRI total** (*p*-value)	**NRI event** (*p*-value)	**NRI non-event** (*p*-value)	**IDI** (*p*-value)
Core + ApoJ	4.08 (0.043)	0.29 (0.105)	0.00 (1.000)	0.29 (0.005)	0.03 (0.085)
Core + ApoL1	3.95 (0.047)	0.26 (0.144)	0.04 (0.773)	0.22 (0.037)	0.02 (0.146)
Core + ApoJ + ApoL1	7.53 (0.023)	0.39 (0.026)	0.08 (0.562)	0.31 (0.0023)	0.04 (0.034)

Results of likelihood ratio test, reclassification, and discrimination analyses are shown. The core model includes age, sex, HOMA-IR, plasma triglyceride levels, waist circumference, and the AST/ALT ratio. “Event” refers to cases (MASH), and “non-event” refers to non-cases (non-MASH)

LRT, Likelihood Ratio Test; ΔDeviance, difference in deviance between the core and extended model; NRI, Net Reclassification Improvement; IDI, Integrated Discrimination Improvement

LRT analyses showed that apoJ (ΔDeviance = 4.08, *p* = 0.043) and apoL1 (ΔDeviance = 3.95, *p* = 0.047) each significantly improved model fit, with their combination yielding additional improvement (ΔDeviance = 7.53, *p* = 0.023). NRI confirmed the strongest effect for the combined model (NRI total = 0.39, *p* = 0.026), driven mainly by improved classification of non-MASH individuals (NRI non-event = 0.31, *p* = 0.0023), corresponding to 31% of true negatives correctly reclassified into lower-risk categories compared with the core model. When assessed separately, apoJ showed a greater effect than apoL1, also predominantly in non-MASH individuals (NRI non-event = 0.29, *p* = 0.005 versus 0.22, *p* = 0.037, respectively). Consistently, IDI was highest for the combined model (IDI = 0.04, *p* = 0.034), indicating a 4% increase in discrimination between MASH and non-MASH individuals compared with the core model.

Overall, adding apoJ and apoL1 to the core model significantly improved predictive performance, with the greatest benefit observed in the correct classification of non-MASH individuals.

## Discussion

MASH is a leading cause of liver transplantation, yet no specific biomarkers are available for efficient diagnosis or prognosis. Obesity and metabolic status strongly drive MASH development [3, 4], but metabolic health alone is unreliable for MASH assessment in individuals with obesity, as it is often transient and deteriorates over time. Moreover, MASH itself can worsen metabolic dysfunction, illustrating a bidirectional interaction [4]. In this context, the early diagnosis of advanced MASH is critical for timely intervention in high-risk subjects with obesity [27]. The identification of non-invasive biomarkers that diagnose MASH independently of risk factors and comorbidities is therefore essential.

Circulating apolipoproteins are sensitive to metabolic disturbances and have emerged as promising candidate biomarkers. Altered apolipoprotein concentrations are commonly related to alterations in lipoprotein metabolism and linked to adverse outcomes, including atherosclerosis and MASLD [11], highlighting their potential role in MASH pathophysiology. However, previous research has been limited, mostly focusing on major apolipoproteins such as apoA-I, apoB100 or apoC-III and MASLD [12–15, 28]. Other structurally and functionally distinct apolipoproteins have remained largely underexplored, in part due to the analytical technical issues.

In this study, we quantified plasma concentrations of 14 apolipoproteins in individuals with obesity undergoing bariatric surgery. As expected, those with MASH were more likely to have T2D and dyslipidemia, along with higher HOMA-IR, hypertriglyceridemia, and a lower AST/ALT ratio (< 1)—a recognized marker of MASH severity [29, 30]—despite comparable BMI and lifestyle factors. We identified significant associations between MASH and plasma levels of apoE, apoJ, and apoL1, independent of age and sex. Associations for apoJ and apoL1 persisted after further adjustment for comorbidities such as diabetes, dyslipidemia, and hypertension as well as traditional risk factors, including central obesity (waist circumference), insulin resistance, and hypertriglyceridemia. Importantly, adding apoJ and apoL1 to established risk factors significantly improved MASH prediction, mainly by reducing risk overestimation and improving identification of non-MASH individuals. Clinically, integrating these biomarkers into risk assessment models could refine patient stratification and support more precise decision-making in MASH management. Validation in larger, independent cohorts will be essential to confirm these findings and to determine whether combining apoJ and apoL1 with other biomarkers can further optimize risk prediction and early detection of MASH.

Consistent with the plasma findings, hepatic mRNA expression of APOJ and APOL1 was also higher in MASH, suggesting that circulating concentrations may reflect hepatic expression, although this relationship warrants further validation. Our results are in line with earlier studies linking plasma apoJ and apoL1 with liver fibrosis in patients with hepatitis C [31]. However, when we analyzed an external replication cohort of individuals with obesity, only apoJ was elevated in MASH, whereas apoL1 was not, indicating that associations with apoL1 should be interpreted with caution. Interestingly, apoF, which has been implicated in hepatic lipid metabolism [16], emerged as a potential plasma biomarker for MASH in the external cohort [22], whereas we observed no such association in our discovery cohort. These discrepancies may reflect methodological differences: the external study used untargeted, relative quantification, which provides broad proteome coverage but may lack sensitivity and reproducibility for low-abundance proteins, while our targeted proteomic approach yielded absolute concentrations with higher specificity, accuracy, and robustness [21]. Importantly, both studies consistently identified elevated apoJ in individuals with MASH, underscoring its promise as a biomarker and illustrating the complementary value of targeted and untargeted proteomics in biomarker discovery.

ApoJ (clusterin) is a secreted glycoprotein mainly originating from the liver and brain and transported in plasma by HDL [32, 33]. It has been proposed to exert an atheroprotective role through HDL-mediated reverse cholesterol transport and by enhancing paraoxonase 1 (PON1) activity, which protects against LDL oxidation [34]. Additionally, apoJ functions as a hepatokine, regulating muscle glucose metabolism via a low-density lipoprotein receptor-related protein-2 (LRP2)-dependent mechanism, thereby protecting against insulin resistance and glucose intolerance [35, 36]. However, in hyperlipidemia, HDL-bound apoJ decreases, while total circulating apoJ increases, correlating with triglycerides, cholesterol, and VLDL-C levels [37]. This redistribution from HDL-bound to unbound apoJ in metabolic syndrome may compromise its metabolic and cardiovascular protective functions. In our study, we measured total plasma apoJ without distinguishing its association with HDL. However, based on the aforementioned report [37], non-HDL-bound apoJ would likely predominate in our study population of individuals with obesity, potentially explaining its association with MASH status. Notably, plasma apoJ was the only apolipoprotein in our dataset that showed no correlation with other apolipoproteins or bioclinical variables such as BMI and HDL-C. Nevertheless, it independently predicted MASH status in multivariable logistic regression analysis. Consistent with this observation, a recent study reported upregulation of apoJ in hepatocytes cultured in high-fat medium and in the livers of patients with MAFLD [38]. Moreover, antagonizing apoJ was shown to promote proteasomal degradation of mTOR and alleviate hepatic lipid deposition [38].

ApoL1, although less consistent across cohorts, may also contribute to MASH pathogenesis. Among the six apoL family members [39], only apoL1 is secreted [39, 40] and it is the most extensively studied due to its role in innate immunity against *Trypanosoma brucei*, the parasite responsible for African sleeping sickness [39]. Although apoL1 is widely expressed in human tissues [41–43], the liver is the primary source of circulating apoL1 [40, 44]. In the bloodstream, it is mainly associated with HDL_3_ particles and, along with PON1 and PON3, may contribute to protecting LDL from oxidation [45]. Supporting a potential atheroprotective function, reduced circulating apoL1 levels in patients with familial hypercholesterolemia have been linked to an increased risk of fatal cardiovascular events [46]. However, a separate study on coronary patients with low HDL-C levels found an association between apoL1 levels and hypertriglyceridemia as well as hyperglycemia [47], suggesting a role in impaired triglyceride and glucose metabolism—both key drivers of MASH**.** Consistently, a cross-sectional study showed that individuals with elevated apoL1 levels were more prone to insulin resistance, obesity, hypertriglyceridemia, and low HDL-C [48]. Furthermore, one longitudinal five-year study reported that higher plasma levels of apoL1, along with apoJ, were associated with an increased risk of developing T2D [49].

Among other candidates, apoC-III and apoE have previously been associated with metabolic dysfunction and MASLD [49–54]. In our study, their associations with MASH did not remain significant after adjustment for metabolic risk factors, suggesting that their impact may be mediated through triglyceride metabolism, insulin resistance, and inflammation rather than directly contributing to MASH pathology.

This study has limitations. First, the limited sample size may affect multiple aspects of our findings as it reduces statistical power, making it more challenging to detect potential effects. Additionally, it can amplify random error, leading to greater variability in results and wider confidence intervals. In the multivariable logistic regression analysis, a smaller sample size limits the ability to adjust for potential confounders. Since MASLD and MASH are influenced by various cardiometabolic factors, residual confounding variables may still be present in our analysis.

Second, there was a numerical imbalance between groups, with fewer individuals in the MASH groups compared with the non-MASH groups in both the discovery (54 versus 94) and external (33 versus 127) cohorts. Such disparities further reduce statistical power and weaken group comparisons.

Third, the control group combined participants without liver alterations and those with simple steatosis into a single non-MASH group. Stratified analyses did not show significant differences between each subgroup and MASH (data not shown), likely due to limited statistical power. Nonetheless, the differences observed when both subgroups were pooled suggest that apolipoprotein alterations are more closely associated with progression to MASH rather than with simple steatosis alone.

Fourth, several associations between the 14 measured apolipoproteins and MASH status had marginal unadjusted *p*-values (< 0.10). Although some showed nominal significance before correction for multiple testing, none of the FDR-adjusted p-values (q) reached the conventional significance threshold. This lack of statistical significance after correction may reflect the relatively small sample size, moderate effect sizes, and the stringent penalty imposed by multiple testing. Therefore, these findings should be considered exploratory. The modest odds ratios for apoJ (1.07) and apoL1 (1.60), with confidence intervals approaching unity, indicate that these findings should be interpreted cautiously, as exploratory and hypothesis-generating rather than confirmatory. Replication in larger, independent cohorts is needed before considering their clinical application.

Finally, the cross-sectional design of our study precludes any conclusions about causality or disease trajectory. MASH is a progressive disease, yet our measurements were taken at a single time point. Longitudinal studies, with repeated measurements of apolipoproteins at different disease stages, would provide deeper insights into their role in disease progression.

## Conclusions

This study provides the first targeted quantification of multiple plasma apolipoproteins in MASH. Plasma apoJ is consistently associated with MASH and emerges as a promising biomarker for risk stratification in individuals with obesity. ApoL1 showed complementary predictive value in the discovery cohort, particularly when combined with apoJ, but its association with MASH status was not replicated in the external cohort. These findings suggest that individual apolipoproteins are unlikely to serve as stand-alone diagnostic markers but can enhance prediction when integrated with established clinical risk factors. Incorporating apoJ and apoL1 into a core risk factor model improved model fit, risk reclassification, and discrimination, especially for identifying non-MASH individuals. Further validation in larger, longitudinal cohorts is warranted to support their inclusion in multi-biomarker panels for early detection and monitoring of MASH progression.

## Supplementary Information

Supplementary Material 1.

Supplementary Material 2.

Supplementary Material 3.

## Data Availability

The data supporting this study are available in the article, in Supplementary Information, or from the corresponding author upon reasonable request.

## Abbreviations

ALT: Alanine aminotransferase
Apo: Apolipoprotein
AST: Aspartate aminotransferase
BMI: Body mass index
BS: Bariatric surgery
DBP: Diastolic blood pressure
FDR: False discovery rate
FPG: Fasting plasma glucose
HDL-C: High-density lipoprotein cholesterol
HOMA-IR: Homeostasis model assessment of the insulin resistance index
IDI: Integrated discrimination improvement
LDL-C: Low-density lipoprotein cholesterol
LRP2: Lipoprotein receptor-related protein-2
LRT: Likelihood ratio tests
MASH: Metabolic dysfunction-associated steatohepatitis
MASLD: Metabolic dysfunction-associated steatotic liver disease
NRI: Net reclassification improvement
OR: Odds ratio
PON1: Paraoxonase 1
SBP: Systolic blood pressure
SD: Standard deviation
T2D: Type 2 diabetes
