# Supplementary material for: ApoJ and apoL1 as novel determinants of MASH: a cross-sectional study
Source: Lipids Health Dis. 2025 Oct 14;24:319. doi: 10.1186/s12944-025-02733-0 (PMC12522655; doi:10.1186/s12944-025-02733-0)
Supplement: Supplementary file 1 — Supplementary Material 1. [file 12944_2025_2733_MOESM1_ESM.docx]

# **Supplementary Table 1. Spearman correlations (r_s_) and p-values between plasma apolipoprotein concentrations**

| **r_s_**  **p-value** | **ApoA-I** | **ApoA-II** | **Apo**  **A-IV** | **Apo**  **B100** | **ApoC-I** | **Apo**  **C-II** | **Apo**  **C-III** | **ApoD** | **ApoE** | **ApoF** | **ApoH** | **ApoJ** | **ApoL1** | **ApoM** |
| --- | --- | --- | --- | --- | --- | --- | --- | --- | --- | --- | --- | --- | --- | --- |
| **ApoA-I** | 1 | 0.5799  <0.0001 | 0.1846   0.0247 | -0.0152   0.8541 | 0.1581   0.0549 | 0.1407   0.0881 | 0.1267   0.1250 | -0.0971   0.2403 | 0.2283   0.0053 | 0.0131   0.8740 | 0.0987   0.2325 | 0.0267   0.7475 | 0.1622   0.0488 | 0.2662   0.0011 |
| **ApoA-II** |  | 1 | -0.0598   0.4706 | 0.2236   0.0063 | 0.1555   0.0591 | 0.0879   0.2880 | 0.1536   0.0624 | 0.0616   0.4568 | 0.118   0.1532 | -0.016   0.8472 | 0.0653   0.4306 | -0.044   0.5954 | 0.1117   0.1767 | 0.287   0.0004 |
| **ApoA-IV** |  |  | 1 | -0.2896   0.0004 | 0.0481   0.5614 | -0.047   0.5701 | 0.1103   0.1820 | -0.1067   0.1969 | 0.0474   0.5677 | 0.1106   0.1807 | -0.0962   0.2446 | -0.0377   0.6495 | 0.025   0.7628 | 0.1223   0.1386 |
| **Apo**  **B100** |  |  |  | 1 | 0.2135   0.0092 | 0.2735   0.0008 | 0.1598   0.0523 | 0.1062   0.1991 | 0.1785   0.0300 | -0.1293   0.1174 | 0.2757   0.0007 | -0.0374   0.6519 | 0.025   0.7627 | 0.1288   0.1187 |
| **ApoC-I** |  |  |  |  | 1 | 0.3379  <0.0001 | 0.5055  <0.0001 | 0.1824   0.0265 | 0.5746  <0.0001 | 0.0113   0.8916 | 0.0702   0.3965 | -0.0574   0.4884 | 0.3345  <0.0001 | 0.399  <0.0001 |
| **ApoC-II** |  |  |  |  |  | 1 | 0.4365 <0.0001 | 0.1884   0.0218 | 0.4617 <0.0001 | -0.1375   0.0956 | 0.098   0.2362 | 0.0549   0.5078 | 0.1271   0.1237 | 0.1941   0.0181 |
| **ApoC-III** |  |  |  |  |  |  | 1 | 0.0308   0.7103 | 0.5266 <0.0001 | 0.1113   0.1782 | 0.1869   0.0229 | 0.1483   0.0721 | 0.2496   0.0022 | 0.2995   0.0002 |
| **ApoD** |  |  |  |  |  |  |  | 1 | 0.1183   0.1521 | -0.1644   0.0459 | 0.1553   0.0594 | 0.0896   0.2788 | 0.148   0.0727 | 0.2115   0.0099 |
| **ApoE** |  |  |  |  |  |  |  |  | 1 | 0.0727   0.3801 | 0.0156   0.8510 | 0.0838   0.3114 | 0.3324  <0.0001 | 0.3229   0.0001 |
| **ApoF** |  |  |  |  |  |  |  |  |  | 1 | -0.2098   0.0105 | 0.0436   0.5988 | 0.1568   0.0570 | -0.0313   0.7054 |
| **ApoH** |  |  |  |  |  |  |  |  |  |  | 1 | 0.1048   0.2051 | -0.0776   0.3486 | 0.1197   0.1473 |
| **ApoJ** |  |  |  |  |  |  |  |  |  |  |  | 1 | 0.0492   0.5530 | -0.1161   0.1598 |
| **ApoL1** |  |  |  |  |  |  |  |  |  |  |  |  | 1 | 0.2286   0.0052 |
| **ApoM** |  |  |  |  |  |  |  |  |  |  |  |  |  | 1 |
