# Supplementary material for: ApoJ and apoL1 as novel determinants of MASH: a cross-sectional study
Source: Lipids Health Dis. 2025 Oct 14;24:319. doi: 10.1186/s12944-025-02733-0 (PMC12522655; doi:10.1186/s12944-025-02733-0)
Supplement: Supplementary file 2 — Supplementary Material 2. [file 12944_2025_2733_MOESM2_ESM.docx]

**Supplementary Table 2. Spearman correlations (r_s_) and p-values between plasma apolipoprotein concentrations and bioclinical variables.**

| **r_s_**  **p-value** | **Age** | **Waist** | **BMI** | **FPG** | **Hb1Ac** | **Insulin** | **HOMA-IR** | **TC** | **TG** | **HDL-C** | **LDL-C** | **SBP** | **DBP** | **AST** | **ALT** |
| --- | --- | --- | --- | --- | --- | --- | --- | --- | --- | --- | --- | --- | --- | --- | --- |
| **ApoA-I** | 0.0883   0.2859 | -0.2388   0.0037 | -0.1798   0.0288 | -0.0663   0.4236 | -0.1302   0.1184 | -0.2335   0.0044 | -0.2271   0.0055 | 0.2971   0.0002 | 0.0645   0.4375 | 0.6093  <0.0001 | 0.0539   0.5302 | -0.0822   0.3208 | -0.0547   0.5088 | 0.0649   0.4495 | -0.1097   0.1876 |
| **ApoA-II** | 0.0131   0.8746 | -0.1114   0.1808 | -0.1405   0.0885 | -0.1112   0.1784 | -0.1371   0.1000 | -0.0651   0.4333 | -0.0775   0.3491 | 0.3191   0.0001 | 0.1047   0.2070 | 0.3271   0.0001 | 0.1637   0.0551 | -0.0999   0.2272 | 0.0156   0.8509 | 0.0378   0.6597 | -0.0368   0.6596 |
| **ApoA-IV** | 0.2024   0.0136 | -0.042   0.6150 | -0.159   0.0536 | 0.2988   0.0002 | 0.313   0.0001 | -0.0374   0.6529 | 0.0292   0.7248 | -0.0681   0.4111 | 0.122   0.1410 | 0.0859   0.3011 | -0.1426   0.0952 | 0.1798   0.0288 | -0.0672   0.4167 | 0.0028   0.9736 | -0.0394   0.6366 |
| **ApoB**  **100** | 0.034   0.6817 | 0.0405   0.6277 | 0.0719   0.3854 | 0.0158   0.8485 | -0.1008   0.2278 | 0.0691   0.4058 | 0.0774   0.3497 | 0.3923  <0.0001 | 0.0993   0.2315 | -0.0805   0.3325 | 0.4152 <0.0001 | -0.0307   0.7109 | 0.0705   0.3946 | -0.0475   0.5802 | 0.0588   0.4806 |
| **ApoC-I** | -0.0049   0.9528 | -0.0198   0.8121 | -0.0281   0.7350 | 0.2057   0.0121 | -0.0052   0.9505 | 0.1766   0.0323 | 0.2275   0.0054 | 0.4016  <0.0001 | 0.3581  <0.0001 | -0.0621   0.4548 | 0.304   0.0003 | 0.0968   0.2419 | 0.2112   0.0100 | 0.1157   0.1767 | 0.2398   0.0036 |
| **ApoC-II** | 0.1601   0.0519 | 0.0999   0.2304 | 0.051   0.5381 | 0.0959   0.2462 | -0.0071   0.9323 | -0.0267   0.7480 | -0.0066   0.9368 | 0.2387   0.0035 | 0.262   0.0013 | -0.0923   0.2660 | 0.1653   0.0526 | 0.1199   0.1467 | 0.1321   0.1095 | 0.0458   0.5937 | 0.1407   0.0903 |
| **ApoC-III** | 0.2033   0.0132 | 0.111   0.1824 | -0.0822   0.3208 | 0.2306   0.0048 | 0.2129   0.0101 | 0.1584   0.0554 | 0.2116   0.0098 | 0.1373   0.0960 | 0.5049  <0.0001 | -0.1712   0.0382 | -0.0318   0.7113 | 0.0578   0.4852 | 0.0378   0.6481 | 0.1907   0.0251 | 0.2088   0.0114 |
| **ApoD** | 0.0388   0.6397 | -0.0446   0.5927 | -0.117   0.1566 | 0.0287   0.7288 | -0.0353   0.6737 | -0.0278   0.7379 | 0.0123   0.8824 | 0.0524   0.5271 | -0.1371   0.0978 | -0.0155   0.8526 | 0.1052   0.2197 | 0.1209   0.1431 | 0.0752   0.3635 | 0.0168   0.8447 | 0.072   0.3877 |
| **ApoE** | -0.0172   0.8354 | 0.05   0.5489 | -0.0574   0.4887 | 0.1304   0.1141 | 0.0116   0.8901 | 0.0823   0.3219 | 0.1283   0.1202 | 0.4305  <0.0001 | 0.4408 <0.0001 | -0.0368   0.6581 | 0.2898   0.0006 | 0.0804   0.3312 | 0.0156   0.8509 | 0.1844   0.0304 | 0.2391   0.0037 |
| **ApoF** | 0.0503   0.5435 | -0.0406   0.6262 | -0.0567   0.4934 | 0.1917   0.0196 | 0.1469   0.0779 | -0.0495   0.5518 | -0.0053   0.9486 | -0.139   0.0921 | -0.0675   0.4169 | 0.0206   0.8045 | -0.1558   0.0680 | 0.1199   0.1468 | 0.0128   0.8777 | 0.0844   0.3252 | 0.0522   0.5311 |
| **ApoH** | 0.0739   0.3722 | 0.0294   0.7248 | 0.0736   0.3739 | -0.07   0.3981 | -0.0516   0.5376 | -0.0209   0.8013 | -0.0411   0.6202 | 0.0424   0.6087 | 0.1118   0.1777 | 0.0913   0.2715 | -0.0629   0.4639 | -0.1233   0.1355 | -0.0516   0.5337 | -0.0578   0.5009 | -0.0172   0.8371 |
| **ApoJ** | 0.0639   0.4400 | 0.0655   0.4320 | 0.1173   0.1557 | -0.0347   0.6751 | -0.0633   0.4493 | 0.0255   0.7595 | -0.0058   0.9441 | -0.0823   0.3202 | -0.0636   0.4442 | -0.0353   0.6708 | -0.0696   0.4172 | -0.0471   0.5698 | 0.0115   0.8893 | -0.039   0.6500 | -0.0324   0.6977 |
| **ApoL1** | -0.0661   0.4247 | -0.1447   0.0814 | -0.0715   0.3877 | 0.1196   0.1478 | 0.0239   0.7755 | 0.0608   0.4648 | 0.0928   0.2619 | 0.1528   0.0638 | 0.1205   0.1459 | 0.0206   0.8048 | 0.1466   0.0862 | 0.0878   0.2889 | 0.1941   0.0181 | 0.182   0.0326 | 0.2234   0.0067 |
| **ApoM** | 0.069   0.4049 | -0.1186   0.1540 | -0.2271   0.0055 | -0.0025   0.9761 | 0.0205   0.8071 | -0.0772   0.3525 | -0.074   0.3713 | 0.2879   0.0004 | 0.1357   0.1012 | 0.311   0.0001 | 0.1255   0.1425 | -0.1087   0.1884 | -0.106   0.1998 | 0.1032   0.2285 | 0.1282   0.1229 |

ALT, alanine aminotransferase; AST, aspartate aminotransferase; BMI, body mass index; DBP, diastolic blood pressure; FPG, fasting plasma glucose; HbA_1c_, glycated hemoglobin; SBP, systolic blood pressure; TC, total cholesterol; TG, total triglycerides
