# Supplementary material for: ApoJ and apoL1 as novel determinants of MASH: a cross-sectional study
Source: Lipids Health Dis. 2025 Oct 14;24:319. doi: 10.1186/s12944-025-02733-0 (PMC12522655; doi:10.1186/s12944-025-02733-0)
Supplement: Supplementary file 3 — Supplementary Material 3. [file 12944_2025_2733_MOESM3_ESM.docx]

**Supplementary Table 3. p- and q-values for plasma apolipoproteins concentrations according to MASH status (corresponding to Table 2)**

| **Apolipoproteins** | **p-value**^1^ | **q-value**^2^ |
| --- | --- | --- |
| ApoA-I | 0.116^1^ | 0,232 |
| ApoA-II | 0.768^1^ | 0,827 |
| ApoA-IV | 0.720^1^ | 0,827 |
| ApoB100 | 0.548^1^ | 0,697 |
| **ApoC-I** | **0.083**^1^ | 0,217 |
| **ApoC-II** | **0.083**^1^ | 0,217 |
| **ApoC-III** | **0.038**^1^ | 0,217 |
| ApoD | 0.391^1^ | 0,547 |
| **ApoE** | **0.090**^1^ | 0,217 |
| ApoF | 0.189^1^ | 0,294 |
| ApoH | 0.904^1^ | 0,904 |
| **ApoJ** | **0.093**^1^ | 0,217 |
| **ApoL1** | **0.025**^1^ | 0,217 |
| ApoM | 0.163^1^ | 0,285 |

^1^ Unadjusted p-value from two-tailed unpaired Student’s t-test (apolipoproteins with p < 0.10 are shown in bold).

^2^ FDR-adjusted p-value (q) using the Benjamini–Hochberg procedure
